# Could Perturbation of Gut Microbiota Possibly Exacerbate the Severity of COVID-19 *via* Cytokine Storm?

**DOI:** 10.3389/fimmu.2020.607734

**Published:** 2021-01-25

**Authors:** Ramachandran Vignesh, Chinnambedu Ravichandran Swathirajan, Zaw Htet Tun, Marimuthu Ragavan Rameshkumar, Sunil Suhas Solomon, Pachamuthu Balakrishnan

**Affiliations:** ^1^ Preclinical Department, Faculty of Medicine, Royal College of Medicine Perak, Universiti Kuala Lumpur, Ipoh, Malaysia; ^2^ Infectious Diseases Laboratory, YR Gaitonde Centre for AIDS Research and Education (YRG CARE), Chennai, India; ^3^ Laboratory Division, Indian Council of Medical Research-National Institute of Epidemiology (ICMR-NIE), Indian Council of Medical Research, Chennai, India; ^4^ Department of Medicine, Johns Hopkins University School of Medicine, Baltimore, MD, United States

**Keywords:** COVID-19 and gut microbiome, COVID-19 inflammation, Severe Acute Respiratory Syndrome Coronavirus-2, gut dysbiosis in COVID-19, microbial translocation in COVID-19, cytokine storm in SARS-CoV-2, microbiome and cytokine storm

## Introduction

The coronavirus disease-19 (COVID-19) pandemic caused by the Severe Acute Respiratory Syndrome Coronavirus-2 (SARS-CoV-2) continues to be a global threat (1). Studies have shown that COVID-19 patients also presenting with gastrointestinal symptoms and SARS-CoV-2 RNA has been detected in stool specimens from patients with severe disease (2–4). One of the pressing scientific questions that remain unanswered is, why the elderly population and those with underlying conditions are at more risk of developing severe COVID-19 complications than the younger population. Gastrointestinal manifestations play a major role in exacerbating proinflammatory cytokines due to disturbance of gut lining by SARS-CoV-2. Here, we discuss the possible role of the gut microbiota and the dysbiosis leading to exacerbated COVID-19 severity and cytokine storm.

## Gut Immunology and Microbiota

The human body is inhabited by a cornucopia of microorganisms, with a rough estimate of about 38 trillion bacteria and gut remains the most densely and diversely colonized organ (5). The gut microbiota play an arterial role in maintaining immune homeostasis. The mucosal immune system, mainly the mucosa-associated lymphoid tissue such as gut-associated lymphoid tissue (GALT) and bronchial-associated lymphoid tissue, is very important since it acts as the primary line of defense against infections (6). GALT includes Peyer’s patches, appendix and isolated lymphoid follicles of the intestinal mucosa. Crosstalk between immune cells of the GALT and gut microbiota is essential to modulate the immune system. The role of gut microbial products in maintaining the balance between regulatory T cell and effector T cell response has been extensively reviewed (7). Furthermore, Short Chain Fatty Acid (SCFA), a product of commensals’ fermentation of fibre-rich diet, is also essential to facilitate the efficient migration of activated T cells to the intestinal lumen by stimulating the CD103+ Dendritic Cells (DCs) (8). Interestingly, the immune factors and cells from the GALT can be transferred to the bronchial-associated lymphoid tissue through various mechanisms thereby offering protection against respiratory infections (9, 10).

When compared to gut microbiota, the understanding of the microbial community of the lung is relatively less. Evidences from several studies insinuate a vital cross-talk between the intestinal microbiota and the lungs, known as the “gut–lung axis”. A plethora of evidence has supported the connection between gut microbiota and lung immunity. Depletion of gut microbiota has also been linked with the functional impairment of the alveolar macrophages with a reduction in reactive oxygen species-mediated bacterial killing capacity (11). Furthermore, the association between antibiotic-induced disturbance of the gut microbiota and improved survival of *Mycobacterium tuberculosis* in the lungs with a high chance of disseminated tuberculosis has also been reported (12). The gut-lung axis is reportedly bidirectional, that microbial components like endotoxins and metabolites from the gut can affect the lung through the bloodstream and in case of lung inflammation, the gut microbiota could be impacted as well (13). In one study, after exposure to the influenza virus, lungs-derived CCR9+CD4+ T cells migrate to the intestine where they cause gut microbiotadysbiosis, resulting in aberrant Th17 response with intestinal injury and gasteroenteritis (14).

## Gut Microbial Metabolites in Modulation of Immune Responses

Microbial metabolites of gut microbial flora, importantly the short chain fatty acids (SCFAs) such as butyric acid and acetic acid are pivotal in modulating the immune and inflammatory responses (15, 16). These SCFAs can discourage the growth of pathogenic microbes by maintaining acidic pH and mucin production in the intestinal environment (17, 18). They are crucial in maintaining the integrity of gut epithelium to contain leakage and translocation. The SCFAs can also act as inhibitors of histone deacetylase (HDAC) and thereby efficiently hampering excessive inflammatory responses by enhancing the numbers and functions of T helper cells, regulatory T cells and Th17 effector cells (19–22). Also, SCFAs like butyrate demonstrate diverse anti-inflammatory functions by activating G protein-coupled receptors (GPCRs), such as GPR43 and by inhibition of the NF-kB pathway (22–24). Through activation of GPR41, SCFAs have been reported to augment CD8+ T cell functions and butyrate can promote differentiation of regulatory T cells and IL-10/18 producing T cells by activating GPR109A (25, 26).

Interestingly, these SCFAs have been found in minute quantities in the lung compartment as well, thereby indicating a possible link between the gut and the respiratory tract (27). Studies have shown that SCFAs aid formation of progenitors of macrophages and dendritic cells (DCs) in the bone marrow and also *via* augmenting the function of T cells, they offer defense against airway inflammation and respiratory tract infections (26, 28). In the pathogenesis of chronic obstructive pulmonary disease (COPD), the hypothesis of gut-liver-lung axis has also supported the fascinating role of SCFA. Apart from the SCFAs, other metabolites of the gut flora such as retinoic acid, niacin, lactate, tryptophan, pyruvate and desaminotyrosine have also been reported to have a role in host immunity (25, 29–33).

## Gut Dysbiosis and Enhanced Gut Permeability

When there is a change in the composition of gut microbiota, owing to various factors, the normal flora are replaced by pathogenic ones and this phenomenon known as gut dysbiosis, is associated with many diseases (34, 35). Studies have shown associations between change in composition of gut microbiota and respiratory infections (36). Several studies have demonstrated the key role of gut microbiota in the pathogenesis of sepsis and ARDS (37).

While the intestinal barrier prevents the translocation of microbes and their harmful products from the gut lumen to systemic circulation, gut dysbiosis could lead to increased permeability of gut barrier (leaky gut). Gut dysbiosis has been observed to correlate with a decrease in the production of the gut bacteria-derived SCFAs such as butyrate thereby leading to increased gut permeability. This facilitates the translocation of microbiota-derived lipopolysaccharides (LPS), particularly from gram-negative bacteria and inflammatory components to general circulation leading to immune activation and inflammatory responses (38). This immune activation primarily happens *via* the toll-like receptor 4 (TLR4) and TLR4 activation in immune cells are known to aggravate the inflammatory processes associated with exacerbation of several clinical conditions. Activation of TLR4 by LPS has been shown to worsen the mortality rates in cases of influenza infections (39).

## Possible Role of Gut Dysbiosis in Pathophysiology of COVID-19 “Cytokine Storm”

The composition and diversity of gut microbiota are affected by various factors, especially ageing. Age-related imbalance of gut microbiota has been well documented and there are reports on the reduced proportion of probiotic strains like *Bifidobacteria*, *Lactobacillus* and bacteria producing SCFAs like butyrate needed for maintaining the integrity of intestinal barrier (40–42). Likewise, there are several evidences supporting the role of gut dysbiosis in ageing-related cardiovascular, renal and metabolic disorders (43, 44).

In case of COVID-19 infection, the disease severity and mortality rates are very high among elderly patients over the age of 65 years, particularly those with pre-existing comorbid conditions such as diabetes, cardiovascular, metabolic and renal disorders (4, 45–49). Immunological aging is reported to be associated with subclinical inflammatory state known as “inflammaging” wherein the Th1 immune responses play a key role, whereas in children there are more Th2 responses, thereby producing less pro-inflammatory molecules. Moreover, alterations in the gut microbiota have been well documented to have an association with respiratory infections (36), inflammatory bowel disease (50), depression (51), type-2 diabetes (52), cardiovascular disease (53) and hypertension (54).

Thus, the high mortality rates among the elderly people and people with underlying medical conditions with COVID-19 possibly point towards the hypothesis that gut microbiota perturbations could influence COVID-19 disease severity and clinical outcome (55, 56). Several studies suggest that mortality associated with COVID-19 are mainly due to the enhanced cytokine and chemokine production contributing to the virally induced hyper-inflammation, referred to as the “cytokine storm” (57–59).

Based on the findings discussed earlier, during conditions like COVID-19, healthy gut microbiota is a requisite to balancing of optimal immune responses preventing an array of excessive inflammatory reactions that could be detrimental. This balance is very crucial that the immune response can have different clinical outcomes and consequences when it is either under reactive or over-reactive.

Bacterial LPS, the Microbe-Associated Molecular Patterns (MAMP) of Gram-negative bacteria can strongly activate the cells of the inflammatory system and the levels of LPS in plasma have been shown to correlate with the degree of intestinal permeability in various conditions. Several studies have demonstrated the association of LPS with T cell activation and elevated pro-inflammatory responses leading to a “cytokine storm” (60).

The chemokine CXCL10 has been observed to play a key role in recruiting of inflammatory cells to the site of inflammation and its role in COVID-19 induced cytokine storm has been shown in both experimental model and patients. A mice-model study using K18 hACE2 transgenic mice infected with SARS-CoV-2revealed significantly pronounced levels of CXCL-10 among those with cytokine storm (61). Studies have demonstrated elevated levels of CXCL10 in COVID-19 patients than healthy controls. Among the COVID-19 patients, the CXCL 10 levels were higher among those required admission to intensive care than those who had less severity (57). This finding supports the possible role of LPS in the severity of COVID-19. Studies also reported increased Levels of IL-1B, IFN-γ, CXCL-10 and CCL2 were also demonstrated as a result Th1 responses (62). Aberrant expressions of a battery of proinflammatory cytokines and chemokines such as IL-6, IFN-α, IFN-γ, IL-1β, IL-12, IL-7, IL-8, IL-9, IL-10, FGF, G-CSF, GM-CSF, IP-10, MCP-1, MIP-1A, MIP1-B, PDGF, IL-18, IL-33, TGF-β, VGEF, CXCL8, CXCL9, CCL2, CCL3, and CCL5 among infected and severe cases of infected patients were also documented (63, 64).

High LPS levels were observed in severe and fatal lung injury cases (65) which signifies that there is indeed a potential implication of LPS in the pathogenesis of the COVID-19 cytokine storm and COVID-19 related microvascular complications which must be investigated. Gut microbiotadysbiosis in some COVID-19 cases may facilitate the translocation of LPS into the portal circulation, which will further stimulate the Kupffer cells residing in the periportal region of the liver, resulting in activation of NF-κB pathway and secretion of TNF-α and IFN-β (66). This effect can cause the hepatic inflammation as well as systemic inflammation especially when LPS reaches the systemic circulation (67, 68). However, in the case of subclinical endotoxemia, that low dose LPS will not be sufficient enough to cause hepatitis but it may cause systemic low-grade inflammation, which can potentiate the effect of cytokine storm and microvascular complications identified in COVID-19 cases. Moreover, proinflammatory effect (IL-8, MCP-1) of low dose LPS on endothelial cells, high sensitivity of vascular smooth muscle cells to the stimulatory action of LPS, the association of endotoxemia with atherosclerosis, and LPS induced insulin resistance effect are considerable factors which could serve as fertile soil for initiating COVID-19 cytokine storm and microvascular injury in COVID-19 cases (69–71). The trigger factor of the cytokine storm may be due to LPS induced CXCL10 expression as discussed above or it may be because of the direct viral effect on the immune system, but the other concept is that low dose LPS can circulate in the plasma in COVID-19 cases with gut dysbiosis and that subclinical endotoxemia can act as a cofactor in facilitating the severe impact of the COVID-19 cytokine storm.

A recent study has reported a significant increase in the permeability of gut epithelial tight junctions in case of severe COVID-19, thereby suggesting a leaky gut situation. The study also noted a steep increase in the level of zonulin, a protein that acts as the physiological mediator of tight junction permeability in the digestive tract. Interestingly, the elevated levels of zonulin were observed to be a marker for increased mortality in severe COVID-19 cases. Measurement of LPS-binding protein, a marker of inflammation also revealed a significant increase among severe COVID-19 cases than milder cases. These findings support the association between severe COVID-19 and gut permeability and microbial translocation (72).

## Impact of SARS-CoV-2 Infection on Gut Microbiota

It is interesting to note that the gut-lung axis crosstalk could imply the impact of SARS-CoV-2infection on the quality and composition of gut microbiota as well. Studies have demonstrated alterations in the abundance and composition of fecal bacteria in COVID-19 patients compared to healthy controls. The pattern of the gut microbiota composition was found to be positively correlating with increased expression of IL-18, the proinflammatory cytokine (73).

COVID-19 patients have been reported to have lesser beneficial gut microbiota and harbor more opportunistic pathogens. Interestingly, the severity of COVID-19 was found to correlate positively with the abundant presence of opportunistic pathogens and negatively with an abundance of anti-inflammatory bacterium *Facealibacterium prausnitizii* (66).

A study has documented the presence of a few bacteria like Streptococcus and Bacteroides to correlate negatively with inflammatory cytokines and positive association with a few other groups of gut microbiota thereby hinting at the potential role of gut microbiota in the predisposition of COVID-19 patients to disease severity. Likewise, in patients with COVID-19, perturbation of enteric RNA and DNA viral flora has been reported and the disease severity was found to be associated with the alternations in gut virome (74).

Interestingly, a recent study that analyzed the levels of 50 gut-associated plasma metabolites using systems biology approach, revealed that most of these metabolites were found to be dysregulated during severe COVID-19 when compared to controls and those with mild disease. The study reports significantly decreased levels of citrulline, an amino acid that is an established marker of gut and enterocyte function. Also, the levels of succinic acid, a well-known marker of gut microbial dysbiosis were observed to be increasing during severe COVID-19 (72).

While discussing on the predisposition of only a certain proportion of COVID-19 patients to develop severe disease, it is of significance to address the gut microbiota–mitochondria crosstalk as well (75). Studies have revealed the role of gut microbiota in influencing various mitochondrial functions including inflammatory cascades mediated by metabolites like SCFA and bile acids. Likewise, mitochondrial functions could alter the gut microflora composition and activity by immunomodulation leading to inflammatory responses during viral infections (76, 77).

Various studies have highlighted the role of administering probiotics and metabolites to retain optimal immune responses and prevent excessive inflammatory responses (31, 78, 79). Studies have demonstrated reduced lung damage caused by viral infection, due to enhanced levels of SCFAs in the blood caused by the change of proportion of Bacteroidetes and Firmicutes attained by food with high-fiber content (23, 26, 80).

A meta-analysis of several randomized clinical trials revealed that people taking probiotics had a 2-fold lower risk of developing upper respiratory tract infections. The study also reported a significant reduction in disease severity among the infected population (81). A study involving 479 adults demonstrated that administration of probiotic bacteria with vitamins and minerals minimized the duration of episodes of common cold and also lowered the days with fever (82). Another study involving 1,783 school children showed a reduction in the incidence of respiratory infection caused by influenza virus following consumption of *Lactobacillus* sp. (83). Probiotic bacteria have also been shown to enhance the responses of vaccines against respiratory viral infections and recent studies have pointed in this direction suggesting that maintaining the balance of intestinal microbiota may be beneficial to COVID-19 patients and aid in recovery due to improved immune status (10, 84, 85).

Interestingly, another logical reason for altered gut microbiota could be the extensive antibiotics usage in the management of COVID-19 (86). Antibiotics result in dysbiosis and increase susceptibility to new infections and inflammatory disorders.

**Figure 1:**
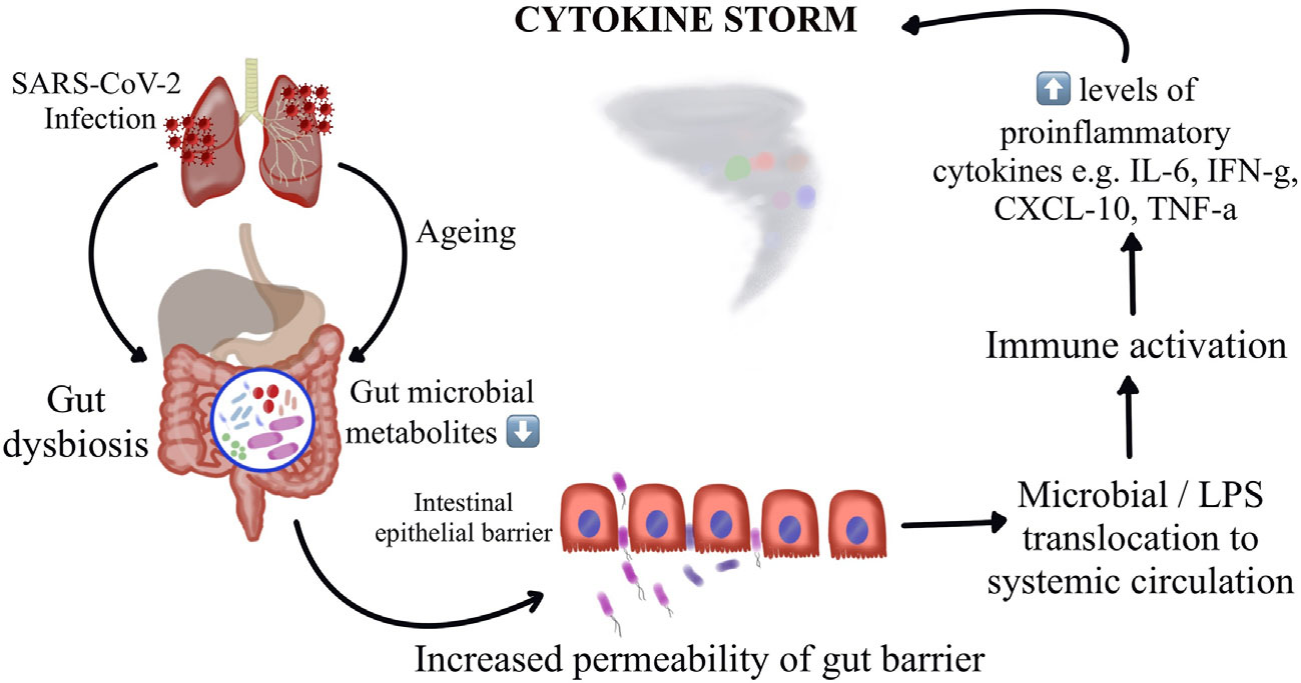
Schematic representation of the proposed hypothesis of gut microbiota perturbation leading to severe COVID-19 by cytokine storm.

Thus, understanding the potential mechanisms by which the gut microbiota regulate host immune and inflammatory responses might offer insights on understanding of the pathogenesis of COVID-19 induced cytokine storm and might throw light on interventions by targeting these microbial florae. A recent Chinese study demonstrates construction of a blood proteomic risk score (PRS) for the prediction of COVID-19 progression to a more severe stage. The study findings revealed a strong association between the gut bacteria, the PRS, and the severity of COVID-19 only in older age groups. Additionally, when analyzing a subgroup of about 301 uninfected individuals over a three-year duration, they observed that the changes in gut microbiota occurred before the alteration could reflect in the PRS, indicating that the gut dysbiosis leads to the protein alterations and not the other way around (56). A study has reported dysbiosis of gut microbiota among hospitalized COVID-19 patients wherein there were reduced levels of probiotic bacteria, a lower proportion of beneficial symbionts and a relatively higher proportion of opportunistic pathogens (66, 87). Interestingly, these variations in the composition of gut microbiota were observed to be correlating with the disease severity. **Figure 1** represents the schematic representation of the proposed hypothesis of gut microbiota perturbation leading to severe COVID-19 by cytokine storm.

## Conclusion

As discussed earlier, the immune-gut interaction being well-balanced and bidirectional, the increased inflammation can lead to leaky gut allowing translocation of bacterial toxins and metabolites to the systemic circulation. This can further worsen the septic state of COVID-19 patients. Earlier studies have demonstrated the association between increased intestinal permeability with sepsis and multiple organ failure (88, 89). Microbial translocation due to poor intestinal integrity ensues a secondary infection and bacterial translocation from the gut to lungs can lead to sepsis and acute respiratory distress syndrome (37). Studies have demonstrated the link between the gut and the respiratory tract and their concerted modulation of immune responses and dysbiosis in gut microbiota impacting the respiratory tract (90). Likewise, through the gut-lung axis, viruses causing respiratory infections in lungs have been known to translocate to other organs *via* systemic circulation. This supports the hypothesis of a disturbed gut microbiota setting stage for disrupted immune homeostasis leading to exacerbation of cytokine storm in COVID-19 patients.

## Author Contributions 

RV, CS, ZT, MR, SS, and PB led the writing of this opinion article. All authors contributed to the article and approved the submitted version.

## Conflict of Interest

The authors declare that the research was conducted in the absence of any commercial or financial relationships that could be construed as a potential conflict of interest.
